# Construction and validation of an aging‐related gene signature for prognosis prediction of patients with breast cancer

**DOI:** 10.1002/cnr2.1741

**Published:** 2022-11-02

**Authors:** Jian Li, Chunling Qi, Qing Li, Fei Liu

**Affiliations:** ^1^ Department of Breast Surgery The Affiliated Taian City Central Hospital of Qingdao University Tai'an City China; ^2^ Postdoctoral Workstation Liaocheng People's Hospital Liaocheng City China; ^3^ Department of Laboratory The Affiliated Taian City Central Hospital of Qingdao University Tai'an City China; ^4^ Department of Pharmacy The Affiliated Taian City Central Hospital of Qingdao University Tai'an City China

**Keywords:** aging, breast cancer, immune, nomogram, TCGA

## Abstract

**Background:**

Breast cancer (BC) is an aging‐related disease. Aging‐related genes (ARGs) participate in the initiation and development of lung and colon cancer, but the prognosis signature of ARGs in BC has not been clearly studied.

**Aims:**

This study aimed to construct an ARGs signature to predict the prognosis of patients with breast cancer.

**Method:**

Firstly, the expression data of ARGs from The Cancer Genome Atlas (TCGA) and Gene Expression Omnibus (GEO) were collected. Then COX and least absolulute shrinkage and selection operator(LASSO) were performed to construct the ARGs prognostic signature. The correlation between the signature and immune cell infiltration, immunotherapeutic response and drug sensitivity were subsequently analysed. The TCGA nomogram was constructed by combining the signature with other clinical features, and was validated by using GEO database.

**Results:**

After LASSO and COX regression analyses, a prognostic signature based on nine ARGs, namely, HSP90AA1, NFKB2, PLAU, PTK2, RECQL4, CLU, JAK2, MAP3K5, and S100B, was built by using the TCGA dataset. Moreover, this risk signature is closely related to immune cell infiltration, immunotherapeutic response, and responses to chemotherapy and targeted therapy. Subsequently, The calibration curve demonstrates that the nomogram agrees well with practical prediction results. The receiver operating characteristic curve and decision‐making curve analysis demonstrate that ARG signature has the better prognosis diagnosis ability and clinical net benefits.

**Conclusions:**

Therefore, the proposed ARG prognosis signature is a new prognosis molecular marker of patients with BC, and it can provide good references to individual clinical therapy.

## 1 INTRODUCTION

Cancer is one of primary causes of deaths in the world. The International Agency for Research on Cancer announced that cancer is a major health concern. In 2020, the number of breast cancer (BC) cases in women exceeded the number of lung cancer cases for the first time. It became the most common cancer in the world and the major reason of female deaths from cancer. BC has been considered as heterogeneous disease,^1^ and it has five different major molecular subtypes, including Luminal A, Luminal B, HER2‐enriched, triple‐negative/basal‐like, and normal‐like according the latest study. Although molecular subtype has greatly contributed to diagnosis and treatment, while some patients still have very poor prognosis. Now, the survival of patients with BC has entered the platform period.^2^ Therefore, new accurate biomarkers should be developed to value the prognosis risks of patients with BC.

The aging of the world's population is currently increasing.^3^ The pathological physiology of aging is caused by many factors through many mechanisms, and the aging process is complicated.^4^ Cell aging is a stress reaction related to human diseases, such as cancer and aging.^5^ Study on aging‐cancer relation is hindered by the complexity and duality, because aging is caused by changes in system and cells. Age is a major cancer risk factor.^6^ Women aged ≥70 have a 1/27 (3.7%) probability of developing BC and hence aging can be attributed as major cause for BC development. Although most old women have a low risk of BC and most old patients with BC died for other reasons, nearly 19 000 deaths were associated with BC among women aged ≥70 every year, and this figure accounts for 47% of all deaths for BC in America. These findings are associated with ongoing oxidative stressors, including aging and invasive BC.^7^

At present, aging‐related genes (ARGs) have been applied to colorectal and lung cancer as a diagnosis or prognosis molecular biomarker.^8^, ^9^ However, ARGs in BC are currently less studied. No accurate ARGs signature has been built to predict the survival rate of patients with BC. Therefore, this study aimed to develop ARG signature for predicting the clinical prognosis of BC according to The Cancer Genome Atlas (TCGA) database. Meanwhile, performances of this signature were validated in the Gene Expression Omnibus (GEO) set. Finally, a risk prediction model nomogram was built based on ARG signature, and this model could predict the prognosis of BC more accurately than simple clinicopathologic characteristics.

## 2 MATERIALS AND METHOD

### 2.1 Data acquisition and processing

Data from transcriptome analysis of patients with BC and relevant clinical information were downloaded from TCGA and GEO. After eliminating cases with less than 30 days of follow‐up period, the tumor samples and clinical data of 1373 cases (1046 cases from TCGA and 327 cases from the GSE20685) were encompassed into analysis. TCGA data were used as the training set, while GSE20685 data were used as the validation set. A total of 307 human ARGs were obtained from the Human Aging‐related Genome Resources (HAGR, https://genomics.senescence.info/download.html). ARG gene list is shown in the Supplementary Material, Table 1. The cBioPortal website was used to evaluate the mutation and variations of copy number in tumor tissues. Both TCGA and GEO are public databases, and all patients involved in them have obtained ethical approval. Information is available and downloaded from relevant data for free to study.

### 2.2 Construction and effectiveness of prognostic ARG signature

To recognize prognosis‐related ARGs, we firstly used univariate COX analysis. The overlapped prognosis ARGs in TCGA and GEO were chosen in subsequent studies. To further narrow range of ARGs, we used least absolute shrinkage and selection operator (LASSO) regression in the training set. And then, multivariate COX regression analysis was performed. Meanwhile, the survival risk signature of patients with BC was established in the training set by using the “glmnet” R package. The risk score formula to predict prognosis of patients with BC is as follows: risk score = mRNA expression level of each ARG × their own coefficient. Patients with BC were separated into the high‐ and low‐risk cluster according to the median risk scores.

### 2.3 Gene changes of ARGs in BC samples and differences of mRNA and protein expression between ARGs and normal tissues

The differences of mRNA expression levels of nine ARGs between tumor and normal tissues were analyzed by BC data in TCGA. The mutations of nine ARGs in the TCGA dataset of BC were analyzed using the online database website cBioPortal(https://www.cbioportal.org/). Differences in protein levels were obtained from CPTAC data on the UALCAN website(http://ualcan.path.uab.edu/index.html).

### 2.4 Function enrichment analysis

The possible mechanism of ARG signature was discovered by the gene set enrichment analysis (GSEA) annotation. To investigate dissimilar molecular mechanisms and pathways among high‐risk and low‐risk groups, we used gene ontology (GO) and Kyoto and encyclopedia of Genes and Genomes (KEGG) enrichment analyses by GSEA 4.1.0 software. The gene sets we used were c5.go.v7.4.symbols.gmt and c2.cp.kegg.v7.4.symbols.gmt. All genes from the breast cancer samples in the TCGA were used to analyze. After 1000 permutations, the gene sets with false discovery rate <0.25 and the normalized *p* < .05 were significantly enriched.

### 2.5 Prognostic value of ARG signature

The median of risk point was marked as the demarcation point to divide patients in the instructing team of TCGA into high‐ or low‐risk groups. The total survival difference between the two groups and the survival differences in various clinical subgroups was measured by Kaplan–Meier analysis. Next, ROC curve, univariate, and multivariate COX regression analyses were used to further assess independent prediction values of the gene signatures in the training set. Finally, the prognostic value of ARG signature was confirmed in the GEO validation set.

### 2.6 Immune cell infiltration analysis

The CIBERSORT algorithm was utilized to calculate infiltration abundances of 22 immune cells. The immune infiltration fraction was calculated by recognizing the cell types. This computation method analyzes the proportion of immune cells based on the characterization of gene expression profile.^10^ Next, the alterations between the low‐ and high‐risk groups in term of infiltration abundance of each immune cell were analyzed through Wilcoxon rank sum test.

### 2.7 Construction and evaluation of nomogram based on ARG signature and clinical factors

To assess the 3‐, 5‐, and 10‐year overall survival (OS) of patients with BC accurately, we built a prognostic nomogram in the TCGA training set based on ARG signature and other major clinical factors. The discrimination ability and prediction accuracy of nomogram were assessed by ROC curve and area under curve (AUC) and the calibration curve. Additionally, decision‐making curve analysis (DCA) was carried out to assess the clinical benefits. Similarly, the performances of the nomogram were evaluated in the validation set.

### 2.8 Comparison of the outcomes of immunotherapy, chemotherapy, and targeted therapy between high‐ and low‐risk groups

The potential clinical effects of immunotherapy in different ARGs groups were assessed using the tumor immune dysfunction and exclusion (TIDE) scoring method. The TIDE, T‐cell dysfunction, and T‐cell exclusion scores of patients with BC in TCGA were calculated and downloaded using the online website (http://tide.dfci.harvard.edu/). The higher predicted TIDE score indicates the higher possibility of immune escape and the lower probability for patients to be benefited from immune checkpoint inhibitor (ICI) therapy. Variances between the high‐ and low‐risk groups in terms of TIDE scores and three ICI gene transcriptional levels were analyzed using Wilcoxon rank sum test. Moreover, a violin plot was drawn for visualizing the differences. Later, patients were divided into high‐ group and low‐risk group according to the median of ARGs risk scores. Differences between two groups in terms of IC50 values of various anti‐tumor drugs (e.g., *Paclitaxel, Doxorubicin, Cisplatin, Carboplatin, Gemcitabine*, *and Lapatinib*) for BC were tested using R package pRRophetic. The Wilcoxon rank sum test was used for statistical analysis.

### 2.9 Statistical analysis

To avoid missing probable ARGs that may be associated with breast cancer prognosis, we set the threshold at *p* < .1 in the univariate Cox analysis. In other analyses, results with *p* < .05 had significantly statistical significance. Data statistical analysis and plotting were completed using R 4.0.2 (https://www.r-project.org).

## 3 RESULTS

### 3.1 Recognition of ARGs related with survival

**FIGURE 1:**
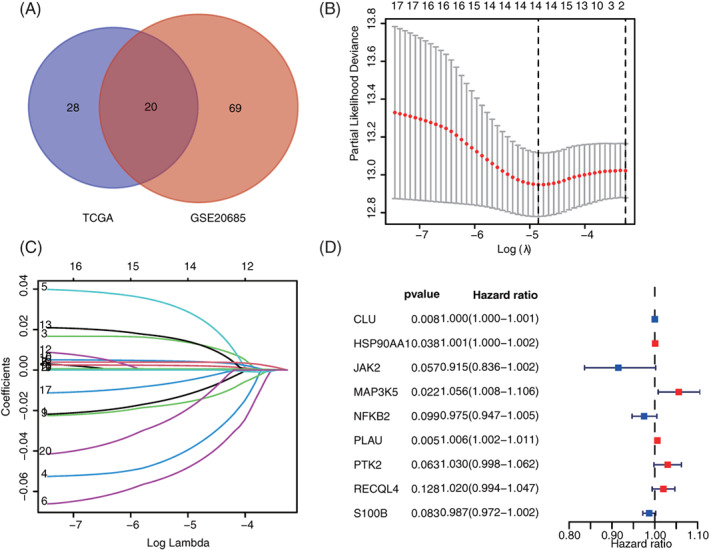
Identification of prognostic signature based on ARGs. (A) 20 common ARGs in TCGA and GSE20685. (B and C) Optimal parameter *λ* was chosen in the LASSO regression model. (D) Nine ARGs (e.g., HSP90AA1, NFKB2, PLAU, PTK2, RECQL4, CLU, JAK2, MAP3K5, and S100B) were screened in multivariate COX regression analysis to build the prognosis risk model. ARGs, aging‐related genes; LASSO, least absolute shrinkage and selection operator; TCGA, The Cancer Genome Atlas

Univariate COX analysis was performed to determine the expression levels of all ARGs in BC. From TCGA and GEO databases, 48 and 89 ARGs were identified to be related with OS of patients with BC, respectively (Supplementary Table 2 and Table 3). Finally, 20 common prognostic ARGs were screened for follow‐up analysis (Figure 1A, Supplementary Table 4). LASSO regression analysis was performed to these 20 ARGs to further decrease the gene number in the model (Figure 1B,C). Later, 14 genes in LASSO were chosen for multivariate COX regression analysis to form the risk signature of ARGs (Figure 1D). Finally, a prognostic signature based on nine ARGs (HSP90AA1, NFKB2, PLAU, PTK2, RECQL4, CLU, JAK2, MAP3K5, and S100B) were built based on 1046 patients with BC in the training set. The prognosis risk scoring formula was established according to the linear combination of expression levels of ARGs and multivariate COX regression coefficient as follows: risk score = 0.000417265 × CLU + 0.000830242 × HSP90AA1 – 0.088314544 × JAK2 + 0.054440541 × MAP3K5 – 0.024912456 × NFKB2 + 0.006408316 × PLAU + 0.02940168 × PTK2 + 0.019917534 × RECQL4 – 0.013127149 × S100B.

### 3.2 Differences in mRNA and protein expression of ARGs in BC

**FIGURE 2:**
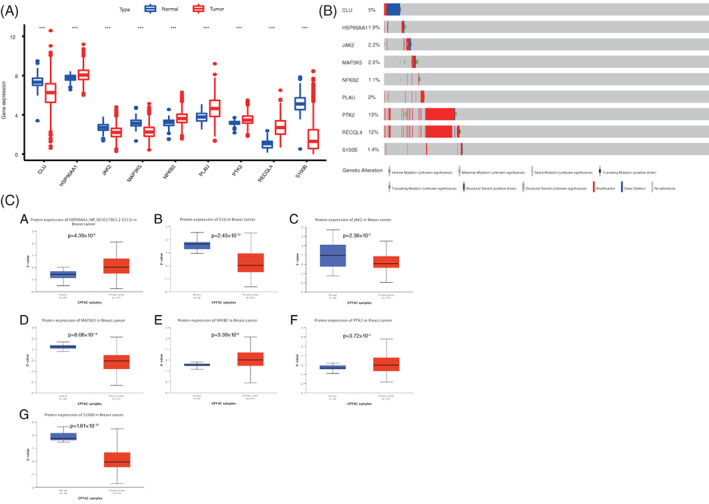
Changes of mRNA of ARGs in BC. (A) Expression differences of ARG mRNA between tumor and normal tissues in TCGA database. **p < .01 and ***p < .001. (B) Genetic changes of ARGs in BC were determined using the cBioPortal database. (C) Differential expression of ARGs at the protein level between tumor and normal tissues in TCGA database. ARGs, aging‐related genes; BC, breast cancer; TCGA, The Cancer Genome Atlas

To determine the mRNA expression levels of nine ARGs, we analyzed the differences of ARG expression levels between normal and tumor tissues in the training set. Results show that the mRNA expression levels of HSP90AA1, NFKB2, PLAU, PTK2, and RECQL4 in patients with BC upregulated significantly, whereas the mRNA levels of CLU, JAK2, MAP3K5, and S100B downregulated sharply (*p* < .001, Figure 2A). The mutations of nine ARGs in the TCGA of BC were analyzed through the cBioPortal database. Results demonstrate that the frequency for gene changes, including amplification, depth missing, and missense mutation, ranges between 1.1% and 13% (Figure 2B). In the protein level, the gene expression of HSP90AA1, NFKB2, PTK2 was upregulated in tumor tissues, and CLU, JAK2, MAP3K5, and S100B were downregulated in the normal tissues (Figure 2C). Of them, the HSP90AA1 was phosphoprotein level, the others were all total‐Protein.

### 3.3 GSEA analysis based on risk groups

**FIGURE 3:**
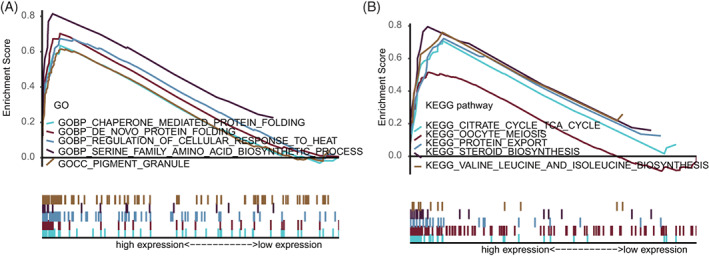
GSEA between high‐ and low‐risk groups (A) GO enrichment analysis between high‐ and low‐risk groups. (B) KEGG pathway enrichment analysis between high‐ and low‐risk groups. GO, gene ontology; GSEA, gene set enrichment analysis; KEGG, Kyoto and encyclopedia of Genes and Genomes

GO and KEGG pathway enrichment analyses were carried out by using GSEA 4.1.0. The c5.go.v7.4.symbols.gmt and c2.cp.kegg.v7.4.symbols.gmt databases were used to further investigate the potential functional mechanisms of different prognosis between low‐ and high‐risk groups in TCGA set. Results demonstrate that ARGs mainly enriched major functions and pathways, such as tricarboxylic acid cycle, oocyte meiosis, protein output, steroid biosynthesis, biosynthesis of valine, leucine, isoleucine, chaperone‐mediated protein folding, cell responses and adjustment to heats, and biosynthesis of amino acids in the serine family (Figure 3A,B). These results might provide references for further understanding of the cell biological effect of ARGs.

### 3.4 Prognostic value of ARG signature in training set

**FIGURE 4:**
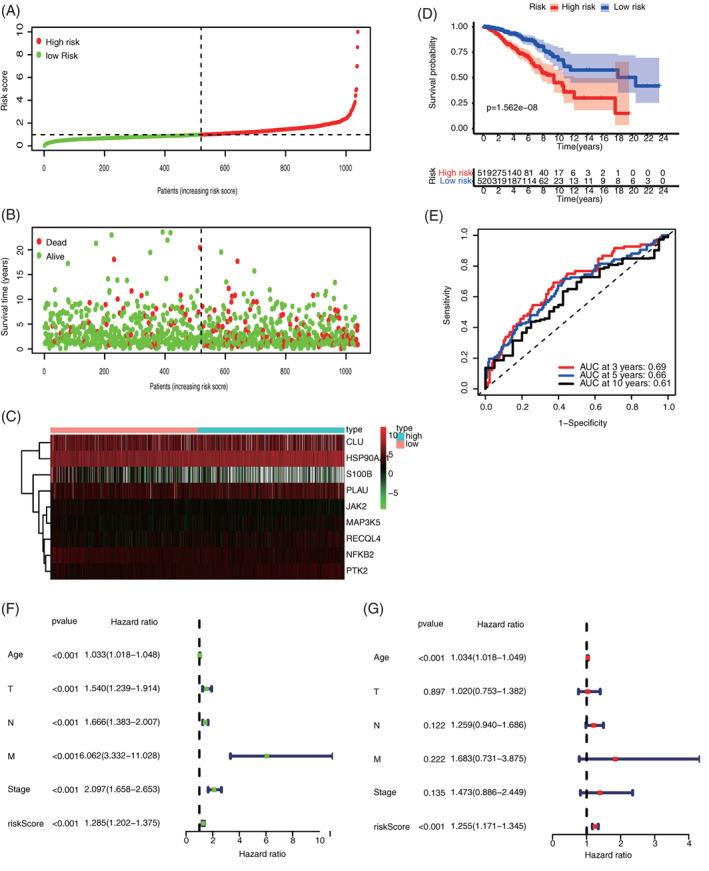
Assessment of the prognostic value of ARG signature in the training set. (A) risk score distribution of all patients; (B) survival state distribution of all patients; (C) heatmap of ARGs expression; (D) comparison of OS curves between high‐ and low‐risk groups; (E) Time‐dependent ROCs of ARG signature; (F) univariate COX regression analysis of signature and other clinical pathological factors; and (G) multivariate COX regression analysis of signature and other clinical pathological factors. ARG, aging‐related gene; OS, overall survival

**FIGURE 5:**
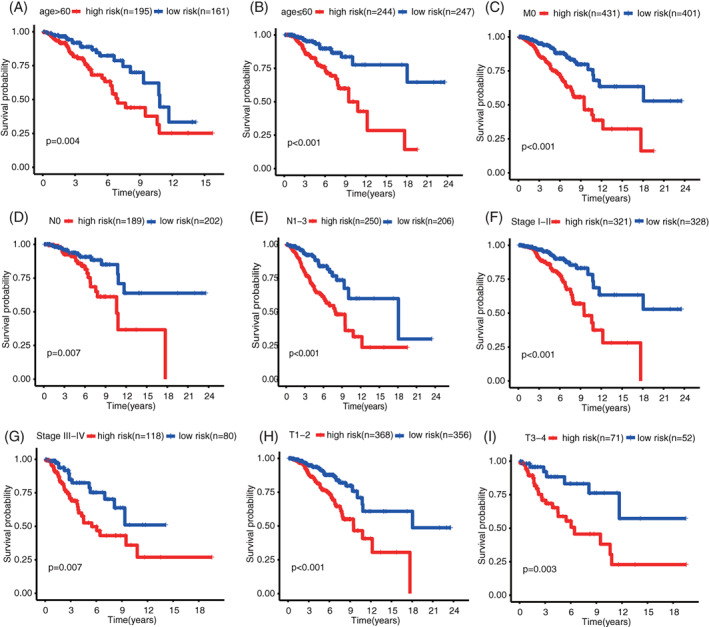
Prognosis evaluation of different BC clinical pathological feature subgroups based on ARG signature; (A) age > 60; (B) age ≤ 60; (C) M0; (D) N0; (E) N1‐3; (F) Stage I–II; (G) Stage III–IV; (H) T1‐2; and (I)T3‐4. ARG, aging‐related gene; BC, breast cancer

The risk marks of all patients in the training set were ordered from low to high, and the patients were divided into the low‐ and high‐score groups according to medians (Figure 4A). The state of life and follow‐up visit time of each patient with BC are shown in Figure 4B. In addition, the heat map of expression spectra of nine ARGs was plotted (Figure 4C). The Kaplan–Meier survival curves of low‐ and high‐risk groups are presented in Figure 4D. The low‐risk group shows significantly higher OS than the high‐risk group (*p* < .001). Next, the accuracy of signature was evaluated by analyzing time‐dependent ROC. The AUC values of 3‐, 5‐, and 10‐year ROC curves in the training set are 0.69, 0.66, and 0.61, respectively (Figure 4E). Besides, univariate and multivariate COX regression analyses were investigated by combining common clinical pathological features, confirming that ARG signature was an independent prognosis predictive factor of BC (Figure 4F,G). Subsequently, the prognostic value of the ARG signature to various clinical subgroups was further validated. Results showed that ARG signature basically can be used to predict the prognosis of all clinical subgroups (Figure 5).

### 3.5 Predicted values of ARG signature in the validation set

**FIGURE 6:**
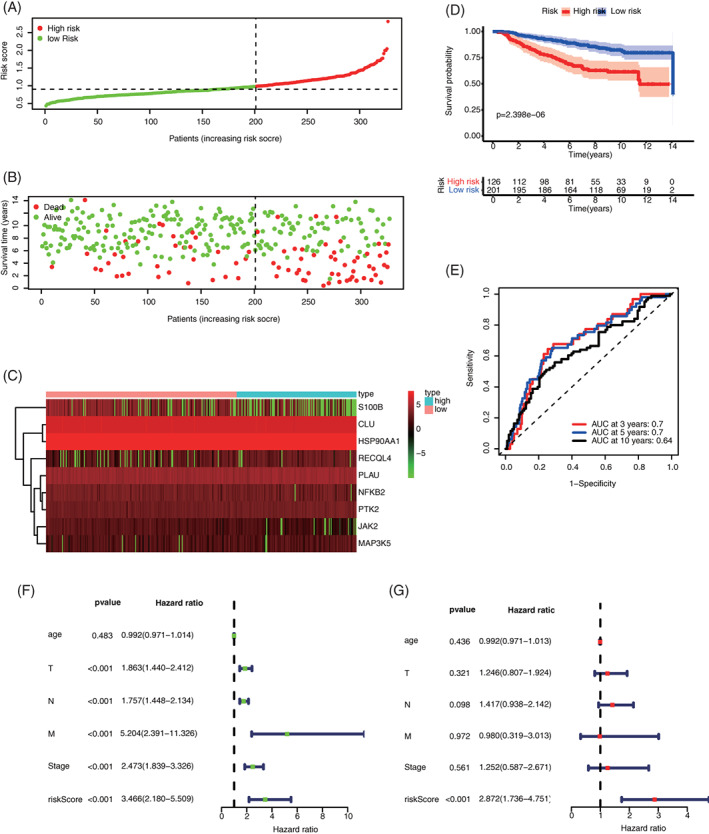
Predicted values of ARG signature in the validation set (GSE20685). (A) Distribution of the calculated risk score; (B) Distribution of the survival state of patients; (C) Heatmaps of ARG expression levels; (D) Comparison of OS curves between high‐ and low‐risk groups; (E) Time‐dependent ROC curves of OS prediction based on ARGs; (F) Univariate COX regression analysis of signature and other clinicopathologic characteristics; and (G) Multivariate COX regression analysis of signature and other clinicopathologic characteristics. ARG, aging‐related gene; OS, overall survival

Similarly, patients with BC in the validation set were divided into high‐ and low‐risk groups according to median risk score. The distributions of risk scores and survival state are shown in Figure 6A,B. Figure 6C showed the heatmaps of nine ARGs in the signature expression difference between high‐ and low‐risk groups. The OS curves of high‐ and low‐risk groups in validation set were compared. The survival of the low‐risk team is drastically higher than that of the high‐risk group. Time‐dependent ROC was also used to assess the accuracy of risk signature. In the validation set, the AUC values of 3‐, 5‐, and 10‐year OS probability were 0.7, 0.7, and 0.64, respectively (Figure 6E). The further univariate and multivariate COX regression analyses proved that ARG signature is an autonomous prediction aspect of BC after the adjustment of clinicopathologic characteristics (Figure 6F, G).

### 3.6 Tumor immune infiltration analysis

**FIGURE 7:**
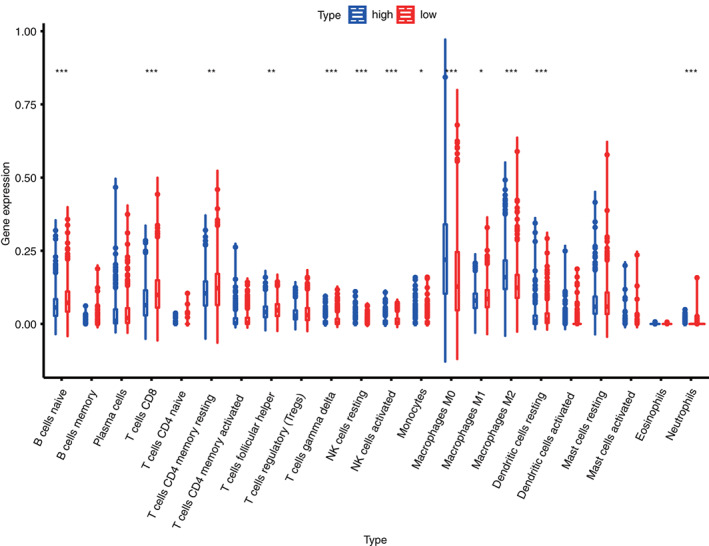
Box plot of the differences in immune cell infiltration between high‐risk and low‐risk groups in the training set

To test relationship between signatures and tumor immune cell infiltration, the infiltration abundances of 22 immune cells of each BC sample from TCGA were calculated by using the CIBERSORT algorithm. The correlations among 22 types of immune infiltration cells are shown in Figure 7. When analyzing the differences between the high‐ and low‐risk assemblies in immune cell infiltration abundances, the low‐risk group showed relatively higher levels in Naive B cells, CD8 T cells, T cells γδ, resting dendritic cells, resting CD4 memory T cells, M1 macrophages, and follicular helper T cells. By contrast, the high‐risk group presented higher levels in NK resting cells, activation of NK cells, M0 macrophage, M2 macrophage, and neutrophils.

### 3.7 Construction of ARG nomogram to forecast individual results of BC

**FIGURE 8:**
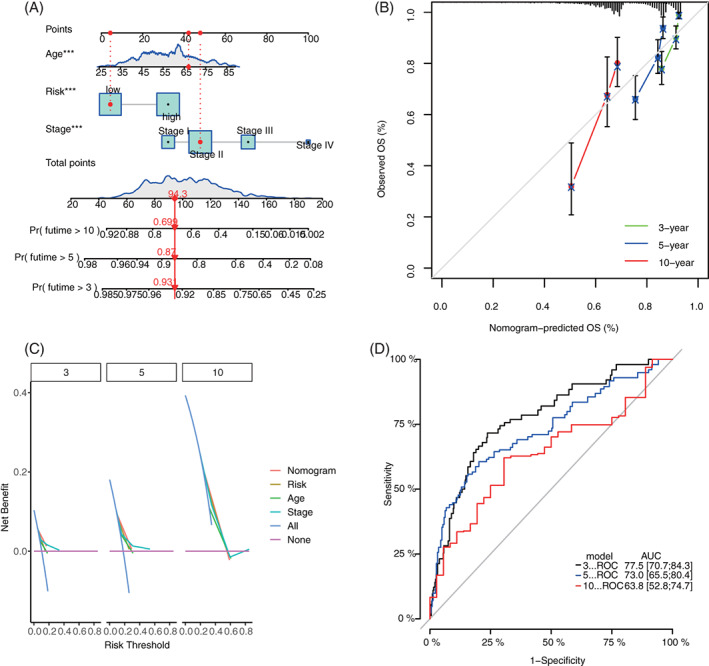
Construction and validation of nomogram model based on clinical features and ARG signature in the training set. (A) Prognostic nomogram combined with age, stage, and ARG signature; (B) 3‐, 5‐, and 10‐year calibration curves of nomogram; (C) DCA of 3‐, 5‐, and 10‐year nomogram; and (D) 3‐, 5‐, and 10‐year ROCs of the model. ARG, aging‐related gene; DCA, decision‐making curve analysis

**FIGURE 9:**
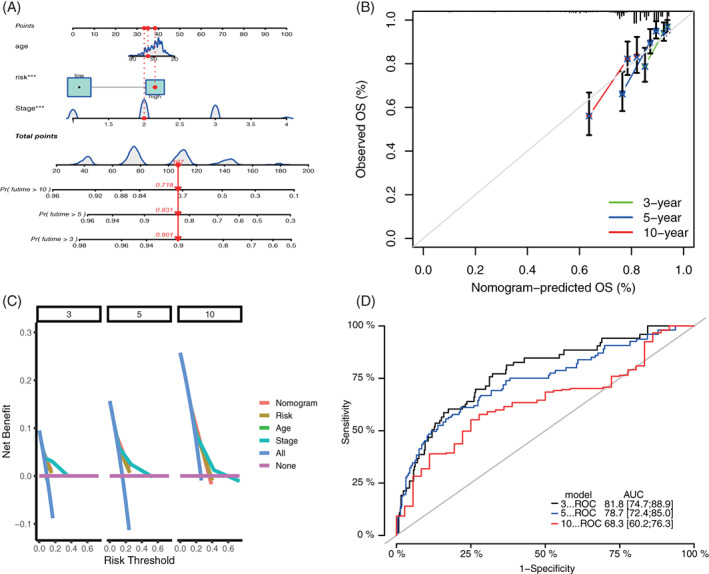
Construction and validation of nomogram model in the validation set. (A) Prognostic nomogram combining with age, stage, and ARG signature; (B) 3‐, 5‐, and 10‐year calibration curves of nomogram; (C) DCA of 3‐, 5‐, and 10‐year nomogram; and (D) 3‐, 5‐, and 10‐year ROCs of the model. ARG, aging‐related gene; DCA, decision‐making curve analysis

Age‐associated gene signature as well as two other valuable clinicopathologic characteristics (age and stage) was chosen to construct the prediction nomogram. This nomogram is an instinctive visualization of the model based on training set of TCGA, and was used to predict the 3‐, 5‐, and 10‐year survival probabilities (Figure 8A). Moreover, it was validated by the same method in GEO (Figure 9A). To further evaluate the prediction performances and clinical application values of nomogram, we compared the calibration curve and DCA. In both training and validation set, the calibration curve of nomogram proves the high consistence between prediction values and practical values (Figures 8B and 9B). Besides, DCA proves that the constructed nomogram model has higher net benefits than clinical predictions based on other clinicopathologic characteristics (Figure 8C and Figure 9C). The 3‐, 5‐, and 10‐year ROCs proved the good diagnosis efficacy of the nomogram (Figures 8D and 9D).

### 3.8 Response differences of high‐ and low‐risk patients to immunotherapy, chemotherapy, and targeted therapy

**FIGURE 10:**
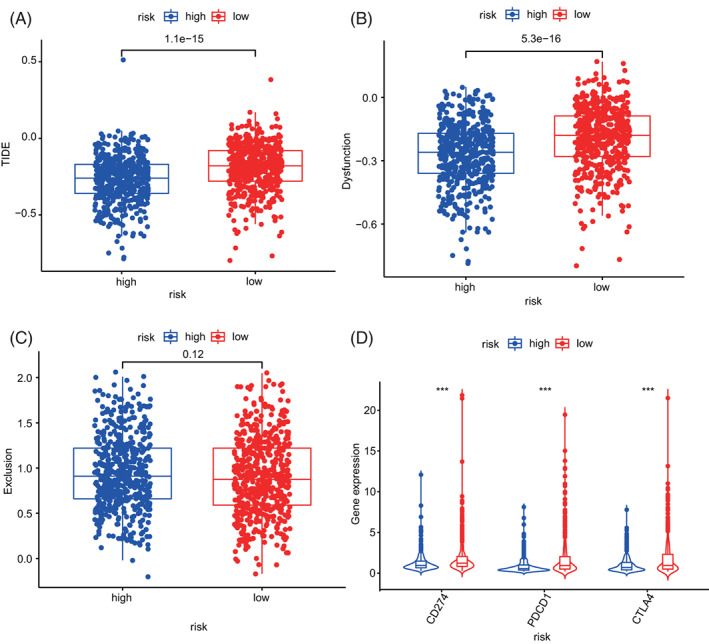
Comparison of immunotherapy prediction scores between high‐ and low‐risk groups

**FIGURE 11:**
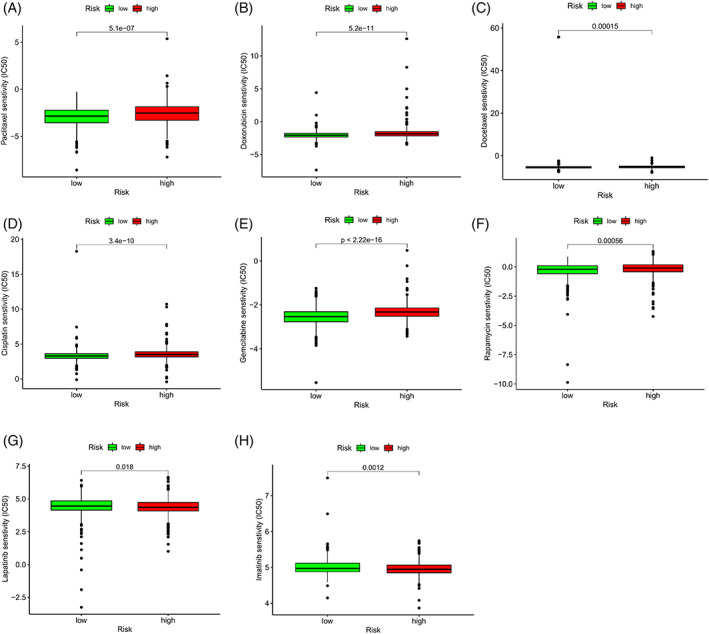
Differences of sensitivity between the high‐ and low‐risk groups to drugs of chemotherapy and targeted therapy. IC50, 50% inhibiting concentration

Results demonstrate that the high‐risk group has lower TIDE scores and T cell dysfunction scores than the low‐risk group (Figure 10A,B). However, no obvious dissimilarities were detected between the two collections in terms of T cell depletion scores (Figure 10C). Generally, the higher TIDE score indicates poor immunotherapy effect. This phenomenon reflects that the high‐risk group may gain more benefit from ICI treatment while the low‐risk group obtain little. In the present study, expression differences of three major clinical ICI genes (CD274, CTLA4, and PDCD1) between the high‐ and low‐risk groups were further studied. Compared to the high‐risk group, the ICI mRNA expression level increased in the low‐risk group (Figure 10D, *p* < .05). These results demonstrate that ARGs are prediction biomarkers of clinical immunotherapy. Besides, the alteration of high‐ and low‐risk groups in terms of sensitivity to chemotherapy and targeted therapy were assessed. Results showed that in contrast to the high‐risk group, the low‐risk group is more delicate to chemotherapy medicines, such as *Paclitaxel, Docetaxel, Doxorubicin, Carboplatin, Gemcitabine*, *and Rapamycin* (Figure 11A–F, *p* < .001) but it is less sensitive to targeted therapy drugs of *Lapatinib* (*p* = .018) and *Imatinib* (*p* = .0012, Figure 11G,H). Therefore, ARG signature is a potential sensitive prediction factor of chemotherapy and targeted therapy.

## 4 DISCUSSIONS

BC is a disease with very strong heterogeneity. Although it has clinical TNM staging system and four molecular subtypes, the discrimination remains unsatisfying, and it cannot predict the prognosis of patients with BC completely. The molecular signatures and prognostic markers of BC have been widely studied,^11^, ^12^, ^13^ but limited studies have focused on the clinical benefits. And the vital function of aging process in BC is still not clear. Therefore, exploring prognostic signature of ARG is vital to understand the function of aging process in BC. There are currently two studies similar to ours,^14^, ^15^ The difference in method was that the ARGs we used to construct the signature were associated with survival overlapped both in the TCGA training set and the GEO validation set, and this method was the most accurate and convincing, while the other two study was only used the TCGA training set to construct the signature which were differed from the method of our study. And our study closely linked prognostic signature to immune, clinical treatment (including chemotherapy, immunotherapy, and targeted therapy) to elaborate the associations with clincal traits, the other two studies were not sufficiently linked to clinical treatment. The difference in result was that our study differs from the other two close studies in that we constructed a prognostic signature containing nine genes overlapped between TCGA and GEO databased, however the other two studies were one with six genes and one with 10 genes only used the TCGA database. Our study analyzed the correlation between the signature and immune cell infiltration, and the sensitivity of immunotherapeutic response, multiple targeted and chemotherapeutic agents, the other two studies were insufficient.

After univariate Cox, the LASSO regression and multivariate COX regression analysis. a molecular prognostic signature built on nine ARGs (e.g., HSP90AA1, NFKB2, PLAU, PTK2, RECQL4, CLU, JAK2, MAP3K5, and S100B) was developed to evaluate the influences of ARGs on the prognosis of BC. The risk score calculated based on the ARG signature can effectively predict the survival of breast cancer patients and guide clinical individualized treatment. Besides, a prognostic nomogram regarding to the ARG signature was built to provide references for clinical decision‐making. This nomogram shows good efficacy in both the training and validation set. Results showed that this signature has good prediction capacity to the survival rate of patients with BC, and it is an independent prognosis factor. Next, this ARG signature was employed to predict the responses in both the high‐ and low‐risk collections to chemotherapy, targeted therapy, and immunotherapy. The low‐risk family is more prone to chemotherapy than the high‐risk one. This finding partially interprets the poor prognosis of high‐risk group considering that these patients might be insensitive to chemotherapy. However, the therapeutic effect of multiple drugs for targeted therapy and immunosuppressors in the low‐risk assembly might be poorer than that in the high‐risk one. To sum up, high‐risk patients might obtain more benefits from targeted therapy and immunotherapy in the future. This finding has to be validated in further clinical patients.

To elucidate the mechanism by which gene mutations affect gene expression, we performed the mutation analysis. In our study, these ARGs were all statistically significant in gene expression between breast cancer and normal tissues, and all had amplification mutations in BC, with PTK2 and RECQL4 being the most prominent. This is consistent with previous reports.^16^, ^17^ This indicates that these genes we used to construct the signature may all have biologically meaningful in BC and have an impact on the occurrence of BC. Most genes in the proposed ARG signature are closely related with the initiation and development of tumor. HSP90AA1 encoded HSP90A, which is a highly conservative chaperone in eucaryon, and it is essential to malignant transformation and development.^18^ It is highly expressed in many malignant tumors, including BC, endometrial cancer, and ovarian cancer. It also inhibits cell apoptosis to promote tumor formation through the steady mutation‐type p53 compounds, and it is an emerging target point of tumor treatment.^19^ As a pleiotropic transcription factor, NFKB2 exists in almost all kinds of cell types and plays vital roles in signal transduction events. These events are triggered by many incentives related with many biological processes including inflammation, immunity, cell differentiation, cell cycle, tumor initiation, and cell apoptosis. NFKB2 has frequent mutations in cancers, and it is related with abnormal TNF signal transduction and tumor diseases.^20^ Moreover, NFKB2 is a target of MYC suppression. NFKB2 deficiency can accelerate the development of lymphoma in Eμ‐myc transgenic mice.^21^ NFKB2 can be used as the prognostic marker of BC.^22^ PLAU encodes a secretory serine protease that transforms plasminogen into plasmin. The overexpression of PSMC2 promotes initiation and development human BC by regulating PLAU.^23^ Protein tyrosine kinase 2 (PTK2) is a type of non‐receptor protein tyrosine kinases, and it is also called adherent spot kinase (FAK). It mediates the signal transduction released by integrins and growth factor receptors. It is overexpressed in several human tumors, including BC.^24^ The overexpression of PTK2, as the key regulator of adhesion and motion, is related to the increased potentials of metastasis. The expression and activity of PTK2 are upregulated in many tumors, and they are related with poor prognosis of patients. Moreover, it promotes stem cell signature and tumorigenicity of hepatocellular carcinoma by activating the Wnt/β‐Catenin signal transduction.^25^ RECQL4 encoded protein is a DNA helicase belonging to the RECQ helicase family. DNA helicase disengages the double‐stranded DNA into single‐stranded ones and might regulate chromosome segregation. The mRNA expression level of RECQL4 increases significantly in BC and suppresses the expression of RECQL4 can inhibit proliferation of BC cells.^17^ Clusterin (CLU) is a protein‐encoded gene. Secretory Clusterin (sCLU) is an extracellular molecular chaperone, and it is related with DNA repair, cell period regulation, deaths of apoptotic cells, and initiation of tumors. sCLU is an interesting gene because of its significances in cancer progression and BC development. It has strong anti‐apoptosis activity and promotes treatment in most BC treatments.^26^ JAK2 gene coding is non‐receptor tyrosine kinases, and it plays the core role in the signal transduction of cytokines and growth factors. The mutation of JAK2 is related to many inflammatory diseases and malignant tumors. JAK2 promotes the growth of BC cells. JAK2 inhibition can constrain the proliferation of BC cells and promote their apoptosis.^27^ MAP3K5 is a member of mitogen activated protein kinase (MAPK) signal cascades and it is activated by various stress stimuli.^28^ MAP3K5 in BC has not been studied. S100 calcium binding protein B (S100B) is a protein‐coding gene, and its expression in BC can be used as the predictive marker of cancer metastasis^29^ and the serum marker of endocrine‐resistance BC.^30^

To further understand the underlying mechanism of influences of ARGs on patients with BC, all patients were divided into the high‐ and low‐risk group based on the median of ARG risk value. Meanwhile, GSEA was carried out. Results show that ARGs mainly participate in major pathways that influence BC, including cell energy synthesis, synthesis of amino acid and steroid, cell proliferation, and division. The aging of immune cells, which occurs gradually in the aging process, is related with the decreased immunological surveillance, and it might be a risk factor of many aging‐related diseases (including cancer).^31^ However, the mechanism in which ARGs regulate immune cell infiltration in BC remains unknown. Therefore, the association of ARGs with immune cell infiltration was investigated in the present study. Results show that high‐risk patients with BC show high expression levels of NK resting cells, NK cells, M0 macrophage, M2 macrophage, T cells γδ, resting dendritic cells, resting CD4 memory T cells, M1 macrophages, and follicular helper T cells. This finding explains why low‐risk patients have better prognosis than high‐risk ones. The high level of M0 macrophages in both low‐ and high‐risk patients may suggest that aging may be closely related to macrophage infiltration. Macrophages (M0) can be induced to polarize into M1, M2 type macrophages. M1 and M2 types have opposite effects and M1 is higher in low‐risk and M2 is higher in high‐risk group, therefore the infiltration patterns of macrophage in the high and low risk groups are different. However, there is some limitations and lack of experimental validation in the study. In the future, more independent BC sets and experiments are needed to validate the constructed ARG signature and mechanism to prove the clinical applicability of ARGs. Overall, a BC prognosis prediction nomogram which combines this ARG signature and other clinical features was constructed and validated. This model can be further employed as a feasible and reliable tool to forecast prognosis of patients with BC.

## 5 CONCLUSIONS

According to the TCGA data set, a novel signature built on nine ARGs for BC prognosis and prediction was constructed and validated. This ARG signature may offer feasible and effective prognosis markers for BC therapy and treatment response prediction.

## AUTHOR CONTRIBUTIONS

**Jian Li:** Conceptualization (lead); data curation (lead); methodology (lead); resources (equal); writing – original draft (equal). **Chunling Qi:** Conceptualization (supporting); data curation (lead); formal analysis (equal). **Qing Li:** Methodology (lead); software (lead). **Fei Liu:** Formal analysis (lead); supervision (lead); writing – review and editing (lead).

## CONFLICT OF INTEREST

There is no disagreement of interest to declare concerning the publication of this manuscript among all authors.

## ETHICS STATEMENT

Because both TCGA and GEO are public and open databases and the patients involved in the databases have obtained ethical approval, relevant data for research can be downloaded for free and related articles can be published with no ethical issues and other conflicts of interest or profit.

## Supporting information


**Supplemental Table 1** Human aging‐related genes (ARGs) list

**Supplemental Table 2** 48 ARGs were identified to be related with OS of patients with BC in TCGA cohort

**Supplemental Table 3** 89 ARGs were identified to be related with OS of patients with BC in GEO cohort

**Supplemental Table 4** 20 overlapped prognostic ARGs between TCGA and GEO cohort were screened

## Data Availability

The assessed and analyzed datasets in this work can be obtained for free in the TCGA and GEO database.
