# Supplementary material for: Construction and validation of an aging‐related gene signature for prognosis prediction of patients with breast cancer
Source: Cancer Rep (Hoboken). 2022 Nov 2;6(3):e1741. doi: 10.1002/cnr2.1741 (PMC10026283; doi:10.1002/cnr2.1741)
Supplement: Supplementary file 1 — Supplemental Table 1 Human aging‐related genes (ARGs) list [file CNR2-6-e1741-s003.docx]

| **Symbol** |
| --- |
| GHR |
| GHRH |
| SHC1 |
| POU1F1 |
| PROP1 |
| TP53 |
| TERC |
| TERT |
| ATM |
| PLAU |
| ERCC2 |
| ERCC8 |
| WRN |
| LMNA |
| IGF1R |
| TXN |
| KL |
| E2F1 |
| PTPN11 |
| NFKB2 |
| STAT5B |
| STAT3 |
| STAT5A |
| NRG1 |
| HDAC3 |
| GH1 |
| IL7R |
| IGF1 |
| IGF2 |
| INS |
| NGF |
| IRS1 |
| PTPN1 |
| IRS2 |
| AKT1 |
| PIK3CB |
| NGFR |
| HRAS |
| MYC |
| EGFR |
| ERBB2 |
| INSR |
| NCOR1 |
| NBN |
| JUND |
| IL2 |
| PDGFB |
| EGF |
| IL2RG |
| FOS |
| PDGFRB |
| EPOR |
| SST |
| PRKCD |
| PPARA |
| RET |
| PLCG2 |
| PEX5 |
| TCF3 |
| PARP1 |
| BRCA1 |
| PIN1 |
| PTEN |
| CREBBP |
| HIF1A |
| UBB |
| RPA1 |
| BLM |
| BCL2 |
| S100B |
| VCP |
| POLG |
| IGFBP3 |
| HSP90AA1 |
| NR3C1 |
| EGR1 |
| VEGFA |
| ABL1 |
| BRCA2 |
| TOP2A |
| TOP2B |
| NFKB1 |
| TOP1 |
| RAD51 |
| UBE2I |
| TNF |
| PDPK1 |
| CEBPA |
| CEBPB |
| MXI1 |
| TGFB1 |
| ERCC6 |
| STK11 |
| EP300 |
| APTX |
| PML |
| GSK3B |
| HTT |
| PRKCA |
| SSTR3 |
| HELLS |
| APOC3 |
| EEF2 |
| ERCC3 |
| TERF1 |
| PRKDC |
| CAT |
| ERCC5 |
| AR |
| GTF2H2 |
| XRCC5 |
| PCNA |
| FEN1 |
| FAS |
| TERF2 |
| XRCC6 |
| POLD1 |
| BAX |
| RB1 |
| EMD |
| GRB2 |
| FOXO3 |
| FOXO1 |
| HSF1 |
| XPA |
| MSRA |
| RECQL4 |
| SOD2 |
| SOD1 |
| FOXM1 |
| COQ7 |
| CACNA1A |
| LRP2 |
| AIFM1 |
| UCHL1 |
| APP |
| APOE |
| A2M |
| SNCG |
| PRDX1 |
| PON1 |
| RELA |
| IL6 |
| RGN |
| ATP5O |
| RAD52 |
| TOP3B |
| ERCC1 |
| SIRT1 |
| HDAC1 |
| HSPA9 |
| GPX1 |
| GSR |
| GSS |
| GSTA4 |
| GSTP1 |
| MT-CO1 |
| HSPD1 |
| HSPA1A |
| HSPA1B |
| PCMT1 |
| MAPK8 |
| YWHAZ |
| PTK2B |
| PTK2 |
| IL7 |
| MAPK14 |
| FGFR1 |
| SP1 |
| FLT1 |
| JUN |
| MED1 |
| MAPK9 |
| MAPK3 |
| HMGB1 |
| CCNA2 |
| HMGB2 |
| MAP3K5 |
| TAF1 |
| LMNB1 |
| SDHC |
| FOXO4 |
| HESX1 |
| PIK3R1 |
| BSCL2 |
| AGPAT2 |
| BMI1 |
| EEF1A1 |
| TFAP2A |
| BDNF |
| CREB1 |
| ATF2 |
| TBP |
| APEX1 |
| HBP1 |
| BUB1B |
| PTGS2 |
| HSPA8 |
| SIN3A |
| CDK1 |
| TFDP1 |
| DDIT3 |
| POLA1 |
| MAPT |
| CTGF |
| HDAC2 |
| MAX |
| MXD1 |
| MDM2 |
| SUMO1 |
| H2AFX |
| HOXB7 |
| HOXC4 |
| JAK2 |
| ESR1 |
| LEP |
| LEPR |
| NFKBIA |
| CLU |
| MTOR |
| GHRHR |
| CTNNB1 |
| PSEN1 |
| DLL3 |
| CDKN2A |
| PPP1CA |
| DBN1 |
| NOG |
| ELN |
| ATR |
| UCP3 |
| ZMPSTE24 |
| TP63 |
| UCP2 |
| POLB |
| GCLC |
| GCLM |
| SIRT6 |
| BUB3 |
| RAE1 |
| PMCH |
| MLH1 |
| CSNK1E |
| STUB1 |
| PPM1D |
| CHEK2 |
| PCK1 |
| ARHGAP1 |
| CDC42 |
| ARNTL |
| CLOCK |
| HIC1 |
| PAPPA |
| ADCY5 |
| PPARGC1A |
| GPX4 |
| UCP1 |
| FGF23 |
| EFEMP1 |
| ERCC4 |
| CETP |
| PPARG |
| AGTR1 |
| CISD2 |
| EEF1E1 |
| EPS8 |
| KCNA3 |
| SIRT7 |
| SLC13A1 |
| SOCS2 |
| TPP2 |
| TP53BP1 |
| SIRT3 |
| NCOR2 |
| SUN1 |
| BAK1 |
| IGFBP2 |
| PYCR1 |
| TP73 |
| CNR1 |
| NFE2L2 |
| CDKN1A |
| PDGFRA |
| PIK3CA |
| C1QA |
| CDKN2B |
| EIF5A2 |
| MIF |
| DGAT1 |
| MT1E |
| FGF21 |
| HTRA2 |
| GSK3A |
| NUDT1 |
| IKBKB |
| SQSTM1 |
| CDK7 |
| GRN |
| SERPINE1 |
| SPRTN |
| RICTOR |
| CTF1 |
| TRAP1 |
| TRPV1 |
| NFE2L1 |
| IFNB1 |
| GDF11 |
