# Supplementary material for: Construction and validation of an aging‐related gene signature for prognosis prediction of patients with breast cancer
Source: Cancer Rep (Hoboken). 2022 Nov 2;6(3):e1741. doi: 10.1002/cnr2.1741 (PMC10026283; doi:10.1002/cnr2.1741)
Supplement: Supplementary file 2 — Supplemental Table 2 48 ARGs were identified to be related with OS of patients with BC in TCGA cohort [file CNR2-6-e1741-s001.docx]

| **Tabe 1. Prognosis-related ARGs in TCGA.** | | | | |
| --- | --- | --- | --- | --- |
| **id** | **HR** | **HR.95L** | **HR.95H** | **pvalue** |
| AIFM1 | 1.031453 | 1.007804 | 1.055658 | 0.008876 |
| CLOCK | 1.097608 | 1.022895 | 1.177778 | 0.009616 |
| CLU | 1.000342 | 1.000004 | 1.000679 | 0.047274 |
| CNR1 | 1.076159 | 1.000944 | 1.157027 | 0.047091 |
| DGAT1 | 1.038731 | 1.015142 | 1.062868 | 0.001186 |
| DLL3 | 1.173121 | 1.028251 | 1.338402 | 0.017586 |
| ERCC1 | 0.939567 | 0.89088 | 0.990915 | 0.021669 |
| ERCC5 | 0.830261 | 0.719794 | 0.957683 | 0.010664 |
| FOS | 0.998188 | 0.996332 | 1.000048 | 0.056145 |
| GCLC | 1.057786 | 1.003856 | 1.114613 | 0.035369 |
| GRB2 | 1.003834 | 0.999796 | 1.007889 | 0.062808 |
| GSS | 1.005991 | 0.999272 | 1.012756 | 0.080655 |
| HSF1 | 1.012982 | 0.998872 | 1.027292 | 0.071511 |
| HSP90AA1 | 1.0012 | 1.000524 | 1.001878 | 0.000507 |
| HSPA9 | 1.009157 | 1.003832 | 1.014511 | 0.000734 |
| HSPD1 | 1.002357 | 0.999901 | 1.004819 | 0.059993 |
| IL2RG | 0.980919 | 0.965596 | 0.996485 | 0.016473 |
| IL7 | 0.650245 | 0.420117 | 1.006432 | 0.053459 |
| IL7R | 0.96061 | 0.927177 | 0.99525 | 0.026187 |
| JAK2 | 0.897282 | 0.82496 | 0.975944 | 0.011475 |
| JUN | 0.996338 | 0.992552 | 1.000138 | 0.058872 |
| JUND | 0.996351 | 0.992944 | 0.99977 | 0.036487 |
| MAP3K5 | 1.042663 | 0.992692 | 1.095149 | 0.095463 |
| MAPK9 | 1.052182 | 0.996721 | 1.110729 | 0.065608 |
| MAX | 0.953176 | 0.916367 | 0.991463 | 0.017002 |
| MXI1 | 1.028706 | 1.001744 | 1.056395 | 0.036751 |
| NFKB2 | 0.964863 | 0.938334 | 0.992142 | 0.011918 |
| NFKBIA | 0.984828 | 0.976338 | 0.993391 | 0.000538 |
| NRG1 | 0.704544 | 0.497919 | 0.996913 | 0.047994 |
| PCMT1 | 1.0252 | 1.012729 | 1.037826 | 6.74E-05 |
| PIK3CA | 1.022046 | 1.010801 | 1.033415 | 0.000112 |
| PLAU | 1.005829 | 1.001456 | 1.010221 | 0.008934 |
| PRDX1 | 1.00166 | 1.000271 | 1.003052 | 0.019184 |
| PTK2 | 1.033882 | 1.005707 | 1.062846 | 0.018096 |
| RAD51 | 1.066141 | 0.995549 | 1.141739 | 0.066903 |
| RECQL4 | 1.020234 | 0.996457 | 1.044579 | 0.095917 |
| S100B | 0.985986 | 0.970062 | 1.002173 | 0.089368 |
| SDHC | 1.057021 | 1.008286 | 1.10811 | 0.021301 |
| SERPINE1 | 1.0026 | 0.999526 | 1.005683 | 0.097512 |
| SIRT3 | 0.938089 | 0.872983 | 1.008051 | 0.081603 |
| SIRT7 | 0.900121 | 0.805556 | 1.005787 | 0.063158 |
| SOD1 | 1.002506 | 0.999606 | 1.005414 | 0.090415 |
| SOD2 | 0.988406 | 0.975463 | 1.001521 | 0.082924 |
| STAT5A | 0.972118 | 0.940797 | 1.004482 | 0.090579 |
| TBP | 1.080495 | 1.012906 | 1.152595 | 0.018822 |
| TERF1 | 1.099576 | 0.998833 | 1.210481 | 0.05285 |
| TP63 | 0.935393 | 0.870242 | 1.005423 | 0.069809 |
| YWHAZ | 1.001962 | 1.00014 | 1.003787 | 0.034778 |
