# Supplementary material for: Construction and validation of an aging‐related gene signature for prognosis prediction of patients with breast cancer
Source: Cancer Rep (Hoboken). 2022 Nov 2;6(3):e1741. doi: 10.1002/cnr2.1741 (PMC10026283; doi:10.1002/cnr2.1741)
Supplement: Supplementary file 3 — Supplemental Table 3 89 ARGs were identified to be related with OS of patients with BC in GEO cohort [file CNR2-6-e1741-s002.docx]

| **Tabe 2. Prognosis-related ARGs inTCGA.** | | | | | |
| --- | --- | --- | --- | --- | --- |
| **id** | **HR** | **HR.95L** | **HR.95H** | **pvalue** |  |
| ABL1 | 1.352589 | 1.159133 | 1.578333 | 0.000125 |  |
| AGPAT2 | 1.075419 | 1.007531 | 1.14788 | 0.028853 |  |
| ARNTL | 0.792567 | 0.627056 | 1.001765 | 0.05175 |  |
| BCL2 | 0.928605 | 0.886693 | 0.972498 | 0.00167 |  |
| BLM | 1.147792 | 0.988343 | 1.332966 | 0.07087 |  |
| BRCA1 | 1.166486 | 1.001763 | 1.358296 | 0.047408 |  |
| BRCA2 | 1.655398 | 1.222617 | 2.241374 | 0.001115 |  |
| BUB1B | 1.105242 | 1.021796 | 1.195502 | 0.012479 |  |
| BUB3 | 0.87924 | 0.79027 | 0.978227 | 0.018059 |  |
| CAT | 0.925967 | 0.865725 | 0.990402 | 0.025029 |  |
| CCNA2 | 1.059006 | 1.007019 | 1.113677 | 0.025594 |  |
| CDKN1A | 1.080528 | 1.014252 | 1.151135 | 0.016479 |  |
| CDKN2B | 1.043393 | 0.996797 | 1.092168 | 0.068407 |  |
| CEBPB | 1.127184 | 1.024982 | 1.239576 | 0.013557 |  |
| CLU | 0.962195 | 0.92329 | 1.002739 | 0.067236 |  |
| CSNK1E | 1.144963 | 1.051884 | 1.246278 | 0.001753 |  |
| DBN1 | 1.200366 | 1.113261 | 1.294286 | 2.02E-06 |  |
| DDIT3 | 1.127584 | 1.022835 | 1.243061 | 0.015786 |  |
| E2F1 | 1.067467 | 1.007706 | 1.130772 | 0.026343 |  |
| EEF1A1 | 0.51285 | 0.245025 | 1.073422 | 0.076402 |  |
| EMD | 1.107136 | 1.018731 | 1.203213 | 0.016528 |  |
| EPS8 | 1.121246 | 1.008157 | 1.247021 | 0.034882 |  |
| ERBB2 | 1.01933 | 0.99946 | 1.039596 | 0.056629 |  |
| ERCC5 | 0.842412 | 0.68943 | 1.029339 | 0.093512 |  |
| ESR1 | 0.972499 | 0.95131 | 0.99416 | 0.013099 |  |
| FGF21 | 1.525581 | 0.927107 | 2.510387 | 0.096488 |  |
| FGFR1 | 1.052655 | 0.99872 | 1.109502 | 0.055844 |  |
| FLT1 | 1.137228 | 0.99956 | 1.293856 | 0.050786 |  |
| FOXM1 | 1.048533 | 1.010508 | 1.08799 | 0.011917 |  |
| GDF11 | 1.658802 | 1.164783 | 2.362349 | 0.005023 |  |
| GSTA4 | 1.063207 | 0.995484 | 1.135536 | 0.067974 |  |
| H2AFX | 1.092692 | 1.013337 | 1.178262 | 0.021202 |  |
| HDAC2 | 1.133966 | 1.030623 | 1.247671 | 0.009919 |  |
| HIF1A | 1.205628 | 1.082897 | 1.342269 | 0.00064 |  |
| HSP90AA1 | 1.22399 | 0.999996 | 1.498159 | 0.050005 |  |
| HSPA9 | 1.103344 | 0.993165 | 1.225744 | 0.066922 |  |
| HSPD1 | 1.036512 | 0.993383 | 1.081514 | 0.098167 |  |
| IGF1R | 0.971371 | 0.938561 | 1.005329 | 0.097558 |  |
| IGFBP3 | 1.082201 | 1.0036 | 1.166958 | 0.040036 |  |
| IL2RG | 0.960015 | 0.925712 | 0.995589 | 0.02794 |  |
| IL7R | 0.956412 | 0.915167 | 0.999516 | 0.047538 |  |
| IRS2 | 0.929993 | 0.867785 | 0.99666 | 0.039911 |  |
| JAK2 | 0.879537 | 0.792127 | 0.976593 | 0.016241 |  |
| LEP | 0.987279 | 0.975577 | 0.999121 | 0.03534 |  |
| LRP2 | 0.962991 | 0.941553 | 0.984917 | 0.001027 |  |
| MAP3K5 | 1.113262 | 1.007611 | 1.22999 | 0.034944 |  |
| MAPT | 0.965871 | 0.935206 | 0.997541 | 0.034901 |  |
| MED1 | 1.071032 | 1.030753 | 1.112885 | 0.00045 |  |
| NFKB1 | 0.866918 | 0.773222 | 0.971967 | 0.014397 |  |
| NFKB2 | 0.930156 | 0.874477 | 0.98938 | 0.021507 |  |
| NGFR | 0.968369 | 0.93462 | 1.003337 | 0.07575 |  |
| NUDT1 | 1.144698 | 1.064176 | 1.231313 | 0.000282 |  |
| PAPPA | 1.902003 | 1.137574 | 3.180115 | 0.014228 |  |
| PARP1 | 0.907375 | 0.829653 | 0.992377 | 0.033383 |  |
| PDGFRB | 1.078727 | 1.00194 | 1.1614 | 0.044283 |  |
| PIN1 | 0.890245 | 0.784115 | 1.010739 | 0.072648 |  |
| PLAU | 1.089716 | 1.03759 | 1.144461 | 0.000591 |  |
| PLCG2 | 0.89887 | 0.800319 | 1.009555 | 0.071946 |  |
| POLG | 1.14351 | 1.025617 | 1.274954 | 0.015709 |  |
| PON1 | 0.79416 | 0.610048 | 1.033838 | 0.086773 |  |
| PTK2 | 1.171097 | 1.053673 | 1.301606 | 0.003392 |  |
| PTK2B | 0.873001 | 0.801026 | 0.951443 | 0.001976 |  |
| PTPN1 | 0.960394 | 0.932093 | 0.989554 | 0.008095 |  |
| PYCR1 | 1.095344 | 1.025316 | 1.170155 | 0.0069 |  |
| RAD51 | 1.217843 | 1.078589 | 1.375075 | 0.001467 |  |
| RECQL4 | 1.091253 | 1.030954 | 1.15508 | 0.002603 |  |
| RGN | 0.842212 | 0.72637 | 0.976528 | 0.022932 |  |
| RICTOR | 0.839517 | 0.702492 | 1.003269 | 0.054346 |  |
| S100B | 0.984032 | 0.968478 | 0.999835 | 0.047676 |  |
| SERPINE1 | 1.069439 | 1.023751 | 1.117166 | 0.002581 |  |
| SIRT3 | 0.839987 | 0.746469 | 0.945221 | 0.003786 |  |
| SLC13A1 | 0.479065 | 0.199716 | 1.149144 | 0.09924 |  |
| SOCS2 | 0.94656 | 0.886691 | 1.010472 | 0.099462 |  |
| SQSTM1 | 1.080615 | 1.006085 | 1.160666 | 0.033474 |  |
| STAT3 | 0.863781 | 0.761745 | 0.979484 | 0.022421 |  |
| STAT5A | 0.866627 | 0.783307 | 0.958809 | 0.005511 |  |
| SUMO1 | 0.829928 | 0.676347 | 1.018383 | 0.074182 |  |
| SUN1 | 1.159 | 1.049704 | 1.279677 | 0.003502 |  |
| TERF1 | 1.292286 | 1.076079 | 1.551935 | 0.006053 |  |
| TFAP2A | 0.966854 | 0.929471 | 1.00574 | 0.093841 |  |
| TOP2A | 1.028004 | 0.997977 | 1.058935 | 0.067839 |  |
| TOP2B | 0.835987 | 0.727457 | 0.960709 | 0.011572 |  |
| TP53 | 0.954908 | 0.906149 | 1.006291 | 0.084448 |  |
| UCP3 | 0.874759 | 0.750839 | 1.019132 | 0.086013 |  |
| VCP | 1.144535 | 0.993657 | 1.318324 | 0.061243 |  |
| VEGFA | 1.06486 | 0.997331 | 1.136961 | 0.060105 |  |
| WRN | 0.864287 | 0.742388 | 1.006201 | 0.060072 |  |
| XPA | 0.901821 | 0.79835 | 1.018703 | 0.096522 |  |
| YWHAZ | 1.178322 | 1.052963 | 1.318606 | 0.004247 |  |
