# Supplementary material for: Construction and validation of an aging‐related gene signature for prognosis prediction of patients with breast cancer
Source: Cancer Rep (Hoboken). 2022 Nov 2;6(3):e1741. doi: 10.1002/cnr2.1741 (PMC10026283; doi:10.1002/cnr2.1741)
Supplement: Supplementary file 4 — Supplemental Table 4 20 overlapped prognostic ARGs between TCGA and GEO cohort were screened [file CNR2-6-e1741-s004.docx]

| TCGA | GSE20685 | overlap |
| --- | --- | --- |
| AIFM1 | ABL1 | IL7R |
| CLOCK | AGPAT2 | HSPD1 |
| CLU | ARNTL | PTK2 |
| CNR1 | BCL2 | ERCC5 |
| DGAT1 | BLM | MAP3K5 |
| DLL3 | BRCA1 | JAK2 |
| ERCC1 | BRCA2 | TERF1 |
| ERCC5 | BUB1B | SERPINE1 |
| FOS | BUB3 | NFKB2 |
| GCLC | CAT | PLAU |
| GRB2 | CCNA2 | CLU |
| GSS | CDKN1A | STAT5A |
| HSF1 | CDKN2B | RECQL4 |
| HSP90AA1 | CEBPB | RAD51 |
| HSPA9 | CLU | HSPA9 |
| HSPD1 | CSNK1E | HSP90AA1 |
| IL2RG | DBN1 | S100B |
| IL7 | DDIT3 | YWHAZ |
| IL7R | E2F1 | IL2RG |
| JAK2 | EEF1A1 | SIRT3 |
| JUN | EMD |  |
| JUND | EPS8 |  |
| MAP3K5 | ERBB2 |  |
| MAPK9 | ERCC5 |  |
| MAX | ESR1 |  |
| MXI1 | FGF21 |  |
| NFKB2 | FGFR1 |  |
| NFKBIA | FLT1 |  |
| NRG1 | FOXM1 |  |
| PCMT1 | GDF11 |  |
| PIK3CA | GSTA4 |  |
| PLAU | H2AFX |  |
| PRDX1 | HDAC2 |  |
| PTK2 | HIF1A |  |
| RAD51 | HSP90AA1 |  |
| RECQL4 | HSPA9 |  |
| S100B | HSPD1 |  |
| SDHC | IGF1R |  |
| SERPINE1 | IGFBP3 |  |
| SIRT3 | IL2RG |  |
| SIRT7 | IL7R |  |
| SOD1 | IRS2 |  |
| SOD2 | JAK2 |  |
| STAT5A | LEP |  |
| TBP | LRP2 |  |
| TERF1 | MAP3K5 |  |
| TP63 | MAPT |  |
| YWHAZ | MED1 |  |
|  | NFKB1 |  |
|  | NFKB2 |  |
|  | NGFR |  |
|  | NUDT1 |  |
|  | PAPPA |  |
|  | PARP1 |  |
|  | PDGFRB |  |
|  | PIN1 |  |
|  | PLAU |  |
|  | PLCG2 |  |
|  | POLG |  |
|  | PON1 |  |
|  | PTK2 |  |
|  | PTK2B |  |
|  | PTPN1 |  |
|  | PYCR1 |  |
|  | RAD51 |  |
|  | RECQL4 |  |
|  | RGN |  |
|  | RICTOR |  |
|  | S100B |  |
|  | SERPINE1 |  |
|  | SIRT3 |  |
|  | SLC13A1 |  |
|  | SOCS2 |  |
|  | SQSTM1 |  |
|  | STAT3 |  |
|  | STAT5A |  |
|  | SUMO1 |  |
|  | SUN1 |  |
|  | TERF1 |  |
|  | TFAP2A |  |
|  | TOP2A |  |
|  | TOP2B |  |
|  | TP53 |  |
|  | UCP3 |  |
|  | VCP |  |
|  | VEGFA |  |
|  | WRN |  |
|  | XPA |  |
|  | YWHAZ |  |
